# Use of Foundational Knowledge as a Basis to Facilitate Critical Thinking: Nurse Educators' Perceptions

**DOI:** 10.1155/2022/3736322

**Published:** 2022-02-01

**Authors:** Agnes Makhene

**Affiliations:** Department of Nursing, Faculty of Health Science, University of Johannesburg, Johannesburg, South Africa

Critical thinking frequently involves the ability to interpret information and make informed decisions based on such information, which usually includes foundational knowledge. There is a need in nursing practice for graduates with critical thinking skills to enable them to make informed clinical decisions that will benefit patients. The facilitation of critical thinking has been elusive to many nurse educators. Critical thinking (CT) is not taught in a vacuum, but integration into content has been found to be beneficial in its facilitation. Consideration of foundational knowledge as a basis for facilitation of CT during teaching and learning remains a challenge in nursing education. This paper explored and described the perception of nurse educators on how foundational knowledge can be used as a basis to facilitate CT during teaching and learning. A qualitative, exploratory, and descriptive design that is contextual in nature was employed. A sample of thirteen (*n* = 13) nurse educators was purposively selected, and data were collected through unstructured individual interviews. The collected data were transcribed verbatim and analysed using Tesch's method of qualitative data analysis. The following themes emerged from the analysed data: (i) conducive environment for thinking; (ii) facilitation strategies to stimulate foundational knowledge; and (iii) foundational, conceptual, and procedural knowledge. It was concluded that CT does not take place in a vacuum and therefore foundational knowledge will be used by students in class to answer questions and in clinical settings as they reason about patients' health problems and to come up with relevant and accurate care plans. They will use the foundational knowledge as an anchor upon which they construct new knowledge using their facilitated CT skills.

## 1. Introduction

Foundational knowledge is the original knowledge upon which new knowledge is built. It can take the form of knowledge of a problem, the solution and foundational competencies that include capacity for critical thinking [1]. It further includes the domain knowledge acquired through education and practice experience in a particular field [2]. Foundational knowledge is made up of the facts, theories, principles, methods, skills, terminology, and modes of reasoning that are essential to more advanced or independent learning in an academic discipline [3]. Students in nursing programmes are expected to identify, explain, and apply foundational concepts, terms, and nursing-related theories to practice. It is mandatory for students to have foundational knowledge as a prerequisite for other courses as they progress from one level to another; for example, the foundational knowledge of sociology is required for holistic care of patients [4]. Foundational knowledge is activated as students return to basic knowledge involving clarification of terminology, distinction between related terms, analyses of definitions considering ensuing learning, and contradictions in the application thereof [5].

Foundational knowledge refers to the basic information that students need to solve problems and construct new knowledge [1]. All nursing students learn from the same body of nursing knowledge that allows for greater depth and breadth of foundational knowledge [4]. On the other hand, CT is the intellectually disciplined process of actively and skillfully conceptualizing, analysing, evaluating, and synthesizing information as a guide to belief and action [6]. Facilitation of critical thinking involves the use of foundational knowledge and skills. Nurse educators are required to tailor didactic activities to meet the learning needs of students and outcomes, as well as fostering engagement in CT using foundational knowledge as a base. The challenge is in the understanding that CT does not take place in a vacuum but is in a context of domain-specific foundational knowledge [7]. This raises the question of how foundational knowledge can be used as a basis to facilitate CT.

## 2. Materials and Methods

The researcher used a qualitative, exploratory, and descriptive design that was contextual in nature [8]. The design was found to be appropriate in that the nurse educators were given an opportunity of sharing their perceptions on how foundational knowledge is used to facilitate critical thinking. A purposive sample of thirteen (*n* = 13) nurse educators who taught in the undergraduate Bachelor of Nursing programme participated in the study. The sampling method was appropriate in that the participants gave in-depth information regarding the research topic.

### 2.1. Study Setting

The nursing department is located in the Faculty of Health Sciences at a university in Johannesburg. The research was conducted in a department of nursing, where the interviews took place in each participant's office where the researcher and the interviewee were present.

### 2.2. Participants

A purposively selected sample was approached face-to-face. The participant was selected based on their experience in teaching in higher education and their in-depth knowledge in nursing. The sampling method was suitable in that participants would be able to provide in-depth and meaningful information. The participants consisted of 13 nurse educators whose ages ranged from 30–60 years. Their experience of teaching in higher education ranged from 10–30 years. Four (*n* = 4) held a PhD qualification, while nine (*n* = 9) held a Masters qualification in nursing, also holding an additional qualification in nursing education and registered as such with the South African Nursing Council. Nurse educators who did not meet the inclusion criteria were excluded. The sample size was determined by data saturation which was reached at the tenth participant; however, further interviews were undertaken with the remaining three participants to confirm data saturation [8].

### 2.3. Data Collection Procedure

Data were collected on a date and time determined by the participants in their offices. Unstructured individual interviews were used to collect data from the participants who responded to the following central question: “How can foundational knowledge be used as a basis to facilitate critical thinking?” Follow-up questions were asked based on the participant's response. The participants consented to the use of an audiotape to record the interview to enable the researcher to obtain verbatim transcription of the data and field notes on their nonverbal cues. The interviews lasted for 60–90 minutes. The researcher used communication strategies, such as probing, paraphrasing, and reflecting to ensure the participants do not move off the focus of the study and to elicit the meaning and accuracy of the collected data. The interviews were held in English. The transcripts were taken back during analysis to the participants as a member checking procedure to ensure that the responses were accurately recorded, thereby establishing credibility, confirmability, and dependability of the data [9].

### 2.4. Ethical Considerations

The study was approved by the Ethics Committee of the Faculty of Health Sciences at the University of Johannesburg as a part of a PhD project (14/05/00). Signed informed consent was provided by the participants and participation was voluntary. Their anonymity and confidentiality were ensured in that neither their names nor information were used anywhere in the study. The participants were also made aware of their right to withdraw at any stage from the study without repercussions [10].

### 2.5. Analysis

The researcher and independent coder analysed the data using Tesch's eight-step open coding method of data analysis [11]. Recorded interviews were transcribed verbatim. This involved listening to the tapes and reading each transcript several times to get a sense of the whole and gain accurate information. The researcher wrote ideas as they came to mind. The most interesting and shortest interview was chosen and read through to look for underlying meaning in the information, while continuing to record any thoughts that came to mind in the margin.

After completing this task, a list of all the topics were made and similar topics were clustered together. The researcher then arranged the topics into columns that were assembled into major topics, unique topics, and leftovers. The list was examined against the original data. Abbreviations of topics were done as codes and these were written next to appropriate segments of the text to determine whether new categories emerged. The researcher found the most descriptive wording for the topics and arranged them according to the way they were related to each other. Lines were drawn between categories to show interrelationships. Finally, the researcher decided on the abbreviation of each category and the corresponding alphabetized codes. The material belongs to each category in one place and performed a preliminary analysis and the existing data were recoded.

The transcribed audio-taped interviews, field notes, and Tesch's protocol for data analysis were presented to an independent coder, who was purposively selected based on a PhD qualification and experience in the qualitative research method and data analysis methods. The independent coder analysed data independent of the researcher, after which a consensus meeting was held to compare data analysis and codes, further ensuring credibility and dependability of data analysis and findings. Trustworthiness was established using strategies of credibility through member checking, whereby themes were taken back to the participants to ensure accuracy and prolonged engagement. Dependability was ensured by providing detailed data of the methods used in the study and confirmability through keeping an audit trail. Lastly, transferability was established through the provision of detailed information that will enable prospective researchers to transfer findings to other contexts. The following themes emerged from the categories: conducive environment for thinking, strategies to stimulate foundational knowledge, and the use of critical thinking language.

## 3. Results

Thirteen (*n* = 13) nurse educators participated in the study. There were 10 women and 3 men, with teaching experience from 10–30 years. Their age ranged from 30 to 60 years. Data analysis yielded three themes, namely, (i) conducive environment for thinking, (ii) facilitation strategies to stimulate foundational knowledge, and (iii) foundational, conceptual, and procedural knowledge.

### 3.1. Conducive environment for Thinking

The nurse educator cited the importance of a conducive learning environment in the facilitation of critical thinking. Nurse educator P7 said: “Where there is trust, the learners will feel free to engage in discussions, arguments and sharing of ideas without fear of being judged. They will know that it is ‘ok' to make mistakes.” Another added that it is important to allow the students to engage freely with the classroom activities without fear of being ridiculed or made to feel stupid.

I agree that an environment where there is trust the learners understand that they can challenge their own thinking and that of others, with the teacher included, without fear of victimization is one that is conducive to thinking. (P4)

“Mutual respect between the nurse educator and students is also important, in that it will assure the students that their input is also valued and important. (P1)

…the student must be made aware that in the critical thinking the nurse educator is a co*-learner.* (P2)

The classroom environment needs to be one where there is mutual trust between the nurse educator and students and among students themselves. This trust relationship will make the students feel psychologically safe and interact or voice their opinion [12].

### 3.2. Facilitation Strategies to Stimulate Foundational Knowledge

The nurse educators asserted that teaching strategies that will stimulate retrieval of foundational knowledge and the use thereof in critical thinking should be used. This was supported by the following assertions by the participants:

The educator must use content and teaching strategies that will enable the student to use their foundational knowledge, such as concepts, categories and theories related to a discipline to enable them to use their facilitated critical thinking skills to build domain-specific knowledge. (P1)

Questioning is one of the strategies that can be used to stimulate foundational knowledge that can be used as a base to facilitate critical thinking. (P9)

Nursing discipline-specific knowledge construction depends on the foundational knowledge of subjects such as biological sciences through which critical thinking can be facilitated. (P5)

Foundational knowledge gives students confidence in practice with an understanding that they have a solid base upon which their critical thinking skills are developed. It is used to draw inferences from, deduce, or move inductively from to draw conclusions about the issue at hand through the application of CT. The participants said:

…the ability to use foundational knowledge as a frame reference enables students to adopt the underlying perspectives, paradigms, processes, and methods of inquiry of one or more disciplines, which can in turn enable an interdisciplinary perspective through the application of their critical thinking skills. (P3)

Critical thinking teaching methods, such as argumentation and group discussion should be used to stimulate the students to use their foundational knowledge to construct new knowledge for themselves through their critical thinking skills. (P6)

The facilitated critical thinking skills will enable the students to learn meaningfully by drawing from their foundational knowledge to solve problems in a meaningful manner. (P12)

### 3.3. Foundational, Conceptual, and Procedural Knowledge

Foundational knowledge is used as a trigger to apply CT skills in emerging areas of science, in core courses of nursing science and research methods, prerequisite student knowledge and skills, and in-depth interdisciplinary training in supporting area of nursing content and methods [13]. The participants asserted that:

Foundational knowledge aids the knowledge related to concept used in nursing. (P7)

Using conceptual knowledge also brings into play the use of procedural knowledge that is applied to skills required in practice. (P4)

Foundational knowledge serves as a conception from which the students pull out concepts that they use to analyse, apply knowledge, and make sense of what is being taught. (P2)

It will be difficult for the student to think critically about the taught content if they don't have the necessary nursing and related sciences foundational knowledge. (P1)

The students need to go through a number of schema of foundational knowledge to draw, analyse and arrive at a judgement (CT skills) that determines its relevance and how it can be used to solve a problem. (P8)

## 4. Discussion

The classroom environment should exhibit democratic values such as dialectical dialoguing, negotiating, and consensus-building to enable the students to develop freedom in expressing their views [14]. The students should share their thoughts without fear of ridicule and negative attitudes that hinder CT. According to this principle, for example, an environment where the class rules are agreed upon by the class supports CT. The learning environment climate needs to encourage the students to feel free to take risks and try out new things in the learning area. The student should feel that their attempts to solve problems are respected. The learning area needs to be organized in a manner that makes working together easy for students [7].

The foundational knowledge provides a core set of knowledge that allows the student whose CT is facilitated to identify problems that are important to the discipline and to develop appropriate solutions. Foundational knowledge forms a basis for students to resolve practice problems using discipline-specific knowledge coherently and meaningfully though their taught CT skills [15]. It was asserted [1, 15] that to solve problems specific to the discipline of nursing, the student must be familiar with foundational knowledge from a range of related disciplines that form a framework within which they will develop CT skills [1]. Foundational knowledge is enduring in that it is used repeatedly to solve problems within the discipline by applying CT skills, such as argumentation. As new phenomena within a discipline emerge (for example, new treatment modalities), foundational knowledge provides the basis for the students to apply CT to predict and explain phenomena within the discipline [3]. This type of knowledge is also substantive and is based on sound, innovative, insightful research and is coherent. It is not piecemeal; instead, it is integrated and cumulative. For content to be learnt meaningfully, foundational knowledge provides a set of general principles that allows for the use of CT to scaffold discipline-specific theoretical and practical aspects for problem-solving and decision-making [12].

The above assertion is supported by researchers [5] who cited that basic, foundational learning of facts, theories, formulas, and skills involving a higher level learning associated with, for example, evaluation of knowledge through application of critical skills includes discipline-specific knowledge and skills, as well as discipline- and profession-transcendent competencies (writing and speaking, information literacy, and research). CT from disciplinary and/or interdisciplinary perspectives involves the student's ability to adopt the underlying perspectives and paradigms, processes, and methods of inquiry of one or more disciplines. The ontology of critical thinking refers to knowledge representation [4]. It involves development of intelligent application of domain-specific knowledge. CT includes the intellectual skills associated with high-quality, self-aware thinking, conceptual clarity, logical reasoning, the ability to interpret information from multiple perspectives, and representing others' ideas with integrity [6].

Foundational knowledge in these content areas will provide in-depth training in the content and methods needed for facilitating the CT of students in nursing science [16]. Conversely, the belief is that students' attainment of foundational skills and factual knowledge occur without active engagement in CT. On the contrary, discipline-specific roles require a degree of domain-specific knowledge; therefore, students may choose to use knowledge based on prior educational or professional experience, giving them enough foundational knowledge to construct better arguments and justifications as CT skills [17]. In this instance, the nurse educator can use argumentation as a teaching strategy in order to stimulate dialogue that will force the students to tap into their foundational knowledge as they analyse, evaluate, justify, and pass judgement on the issue under discussion. The educator is required to have the ability and willingness to recognize the importance and value of strong foundational knowledge that provide a basis for facilitating CT [7].

On the other hand, CT reflects the capacity for higher-order thinking, including reflection on one's own thinking, evaluation of information, and hypothetical thinking about alternatives [18]. Students should be encouraged to analyse, compare and contrast, draw inferences, and explain the content using their foundational knowledge. These CT skills are employed by the student to progress to developing functional, conceptual, and procedural knowledge that is applied in practice. Although written tests and examinations are still needed to assess the foundational knowledge comprehensively, integrating assessment of CT skills is essential to determine the competence of a student [18]. Teaching and learning should allow for greater application of foundational knowledge, decision-making, CT, leadership, research utilization, and resource management in clinical practice [7]. Integration of all areas of the discipline affords the opportunity for students not only to provide relevance to practice but also to delineate discipline approaches, whilst integrating foundational knowledge into their facilitated CT to solve problems [19].

CT activities that involve evaluation of cases by students from a variety of disciplines lead to horizontal integration of foundational knowledge that is both intradisciplinary and interdisciplinary in nature [20]. Likewise, CT is more than using a particular skill in an appropriate context; it is both the ability to recognize when a skill is needed and the willingness to apply it. It is highlighting the importance of teaching students how to use their CT skills specifically, CT requires the right knowledge, thinking skills, and the right attitude [6, 16]. Furthermore, developing the attitude, or dispositions, of a critical thinker is an essential component of CT and that many errors occur because people do not think critically, not because they are not able to do so but because they are not disposed to doing so. Therefore, a range of important CT dispositions including a unique focus on planning, mindfulness, and metacognitive monitoring is highlighted [21].

It is, therefore, important for nurse educators to unearth different methods of teaching that engage and encourage students to be actively involved in CT and to make it their daily cognitive tool to enhance their quality of patient care. This challenges the nurse educators to reshape education by adopting instructional strategies that equip students with CT skills, which will empower them to solve practice problem creatively while collaborating with other role players in the multidisciplinary healthcare team in the day-to-day care of patients [12, 20]. This enhances the use of interdisciplinary learning and knowledge construction using critical thinking skills. Given the centrality of self-efficacy to the aforementioned areas of influence on the nursing practice of students and graduates, it is vital to recognize the importance of both self-efficacy and foundational knowledge in the facilitation of CT [22].

In fact, it could be argued that clinical practice lies at the intersection of self-efficacy in CT and knowledge of core concepts of a discipline [15]. Foundational knowledge involves understanding and remembering information and ideas, developing the ability to apply acquired skills, such as performing a complex nursing procedure meant to trigger critical, creative, and/or practical thinking, linking concepts, ideas, and experience. Finally, the student will acquire new skills of an intellectual nature, thereby becoming self-aware and exhibiting attributes of CT, such as intellectual humility, intellectual perseverance, and fairness in judgement among others [13].

Educator-directed reinforcement of independently acquired knowledge to connect foundational knowledge and its application is important. This involves moving towards acquisition of foundational knowledge outside the classroom through the development of self-paced learning content and using class time for active and applied-primarily case-based-learning which provides for acquisition of CT skills [4, 19]. Foundational concepts may be necessary to achieve the full benefit of applied learning, such as analysis of clinical cases. The focus lies in the educator's ability to identifying essential foundational content and condensing foundational knowledge. They need to concentrate on key concepts with emphasis on such knowledge and its application and active knowledge construction as a means for stimulating CT and a deeper sense of learning [23, 24]. Integration involves the blending of foundational knowledge across subject areas (often within the same discipline) that require the students' use of facilitated CT skills to apply that knowledge to problem-solving and decision-making in clinical practice.

Students are usually challenged to use CT to connect different types of foundational knowledge (horizontal integration) and then apply it practically to a real or fictional patient (vertical integration) [1, 17]. The use of foundational knowledge of the discipline in practice should include the application of CT skills [25]. Foundational knowledge involves using propositional knowledge and beliefs concerning the meaning and descriptions of relevant concepts and the relationships between them. It includes multiple factors that are identified as being significant to the teaching and learning of CT, the ontological status of CT, and the purpose of teaching it [18].

Foundational knowledge is what is needed to know which is core content knowledge of the disciplines, information literacy (sometimes called digital literacy), and cross-disciplinary knowledge or synthetic knowledge [2]. The suggestion is that the conceptual knowledge (information) and procedural knowledge (process-based information) used for problem-solving or step-by-step task completion forms the basis for acquisition of domain-specific knowledge [26]. For example, the student who analyses relationships between a respiratory condition and cardiac output and those defined as “other” will need conceptual knowledge of the respiratory and circulatory systems and mechanisms within the two systems to understand their points of convergence or divergence. However, they rely on procedural knowledge of various disciplines to promote critical probing, for example, physiology, physics, and so on. The foundational knowledge will form a basis on which the CT skills within the knowledge parameters are facilitated, developed, and applied to problem-solving [27]. Therefore, it is essential to integrate units forming their existing schema to promote the students' ability to think critically about the presented content. Foundational knowledge provides foundational competencies that include the capacity for CT and the domain or technical knowledge acquired through education and practice experience in a particular field [26].

This means that foundational knowledge includes multiple factors that have been identified as being significant to the teaching and learning of CT, the ontological status of CT, and the purpose of their facilitated CT skills to explore contemporary educational experiences and to promote the fundamental principles of learning [3, 24]. Therefore, it is important that the educator is aware of the importance of the use of foundational knowledge as a basis of facilitating CT skills in students. The student draws from the schema forming their foundational knowledge as a point of reference to think critically. The educator needs to scaffold the foundational knowledge of the students to enable them to use facilitated CT skills to construct new knowledge [20].

## 5. Conclusions

This study has implications both for nursing education in the classroom and clinical setting. The nurse educator and the clinical facilitator need to understand what foundational knowledge is relevant for each level of study, be able to engage the students in such a manner that they are able to retrieve it. Furthermore, they must be able to select appropriate teaching strategies that will facilitate the students' critical thinking skills to enable them to use their foundational knowledge as an anchor upon which they will be able to develop domain-specific and discipline-specific knowledge [20]. Nurse educators need to make use of dialogue that involves questioning and group discussions as facilitation strategies while assessment involves identification of arguments, analysis of arguments, identifying flaws in arguments, evaluating credibility of an argument, and building further argument. These strategies will encourage the students use their meta knowledge consisting of critical thinking to draw and apply on their foundational knowledge which consists of core disciplinary knowledge, cross-disciplinary knowledge, to problem-solving through collaboration with others [2].

It can be concluded that CT cannot be taught in isolation but that foundational knowledge forms a basis for its facilitation. The use of foundational knowledge to facilitate CT as an essential skill in nursing is recommended. This means that foundational knowledge is important in that it forms a knowledge-specific frame of reference that will be used to create new knowledge using their facilitated critical thinking [18]. It is important to trigger cognitive identification of stored knowledge during teaching and learning to enable the student to develop and apply critical thinking skills to construct new knowledge that will be used in real-life clinical settings.

## Data Availability

The data used in this paper are available upon request.
